# Inferring the distribution of selective effects from a time inhomogeneous model

**DOI:** 10.1371/journal.pone.0194709

**Published:** 2019-01-18

**Authors:** Amei Amei, Shilei Zhou

**Affiliations:** 1 Department of Mathematical Sciences, University of Nevada, Las Vegas, Nevada, United States of America; 2 54 Crescent Ave, Apt G, Dorchester, Massachusetts, United States of America; Universitat Pompeu Fabra, SPAIN

## Abstract

We have developed a Poisson random field model for estimating the distribution of selective effects of newly arisen nonsynonymous mutations that could be observed as polymorphism or divergence in samples of two related species under the assumption that the two species populations are not at mutation-selection-drift equilibrium. The model is applied to 91*Drosophila* genes by comparing levels of polymorphism in an African population of *D. melanogaster* with divergence to a reference strain of *D. simulans*. Based on the difference of gene expression level between testes and ovaries, the 91 genes were classified as 33 male-biased, 28 female-biased, and 30 sex-unbiased genes. Under a Bayesian framework, Markov chain Monte Carlo simulations are implemented to the model in which the distribution of selective effects is assumed to be Gaussian with a mean that may differ from one gene to the other to sample key parameters. Based on our estimates, the majority of newly-arisen nonsynonymous mutations that could contribute to polymorphism or divergence in *Drosophila* species are mildly deleterious with a mean scaled selection coefficient of -2.81, while almost 86% of the fixed differences between species are driven by positive selection. There are only 16.6% of the nonsynonymous mutations observed in sex-unbiased genes that are under positive selection in comparison to 30% of male-biased and 46% of female-biased genes that are beneficial. We also estimated that *D. melanogaster* and *D. simulans* may have diverged 1.72 million years ago.

## Introduction

Comparison between silent (or synonymous) polymorphism with amino acid replacement (or nonsynonymous) polymorphism has served as a basis of inferring natural selection for more than 30 years [1]. The original idea of comparison within one species [1, 2] has been extended by Hudson et al. to comparing polymorphisms within species with fixed differences between species [3]. Given aligned DNA sequences from two closely related species, McDonald and Kreitman [4] proposed a statistical test of neutrality for a 2 × 2 contingency table whose four entries are total numbers of silent or replacement polymorphic sites within species and fixed differences between species (see also [5–13]). Application of the statistical test on 30 aligned DNA sequences from the alcohol dehydrogenase gene of three species of *Drosophila* suggested that adaptive fixation of selectively advantageous mutations may have resulted in a statistically significant excess of divergent replacement sites [4]. Rigorous theory underlying the McDonald-Kreitman test was later developed by modeling frequencies of mutant sites as a Poisson random field (PRF) [14]. Within each gene, the model can be applied to polymorphism and divergence data of two related biological species to make statistical inference of various genetic parameters, such as mutation rate, selection coefficient of a nonsynonymous mutation and species divergence time (see also [15–19]). Later, the model has been extended to multiple genes via a hierarchical Bayesian framework [20–25]. Among them, Bustamante et al. [22] proposed a hierarchical Bayesian fixed effects model and application of the model using Markov chain Monte Carlo (MCMC) simulations found evidence of predominantly beneficial gene substitutions in *Drosophila* but detrimental substitutions in the mustard weed *Arabidopsis*. One generalization of the fixed effects model was a rather sophisticated Bayesian random effects model [23] and application of the model to a set of 91 *Drosophila* genes in two species of African populations found that about 95% of nonsynonymous mutations that could contribute to polymorphism or divergence are deleterious and most of fixed differences between species are driven by positive selection [24].

Although the PRF model of Sawyer and Hartl provides an appealing theory for use of polymorphism and divergence data, certain biologically unrealistic assumptions were made for mathematical convenience. In addition to the assumptions of random mating, genic selection, no migration between species, and independence among nucleotide sites, it also assumed that two species have reached mutation-selection-drift equilibrium after divergence, selection coefficients of nonsynonymous mutations at individual locus are constant, and the effective population sizes of the two daughter species and their common ancestor are the same. More recently, efforts have been made to relax these assumptions. For example, Wakeley [26] relaxed the assumption of no migration by studying natural selection and genetic drift in an island model of subdivision and concluded that the inference about natural selection made from DNA polymorphism and divergence data are robust to population subdivision for relatively moderate migration rate. Williamson et al. [27] relaxed the assumption of genic selection by generalizing the PRF model to allow arbitrary dominance relations in a diploid context. Using polymorphism data in a site frequency spectrum form, the generalized model yielded maximum likelihood estimates for both selection and dominance parameters of new mutations. They also used simulations to study the bias in estimates of selection parameters caused by ignoring dominance relations and the results are quite surprising. For frequency spectrum polymorphism data, inference of selection parameters can be strongly biased even for minor deviation from the genic selection model. However, the estimates of selection parameters based on polymorphism and divergence (McDonald-Kreitman) data are nearly unbiased, even for completely dominant or recessive mutations. For the assumption of independent among sites, Bustamante et al. [16] used a PRF model of directional selection at DNA sites to study the power of a likelihood ratio test (LRT) of neutrality for varying levels of mutation and selection as well as the robustness of the LRT to deviations from the assumption of free recombination among sites. Based on their study, the LRT has high power to detect deviations from neutrality but it is not robust to deviations from the assumption of independence among sites; see also [28].

The time equilibrium assumption has been removed in a so called time-dependent PRF model where the selective effects of nonsynonymous mutations within each genetic locus are still assumed to be constant in the model [29, 30]. Application of the time-dependent PRF model to a nuclear and mitochondrial DNA data of 22 sister pairs of birds that have diverged across a biogeographic barrier found temporal differences in divergence times, effective population sizes, and selective coefficients between the taxa that inhabit humid or drier habitats [31]. The model has also been applied to a data set containing the full-length coding region of the rice blast disease resistance gene *Pi-ta* gene from ten rice groups within *Oryza sativa* and the wild progenitor species *O. rufipogon* to estimate speciation and selection [32]. There are other studies where the time-homogeneous assumption was kept but the hierarchical Bayesian fixed effects structure was extended to a Bayesian random effects model in which selective effects of nonsynonymous mutations within individual genetic loci are assumed to follow a normal distribution [23, 24]. In order to obtain accurate estimates of various genetic parameters, it is necessary to build a biologically more realistic model which takes into account the inhomogeneity feature of time as well as the randomness of the selective effects of mutations within genetic loci. In this paper, we present such a model, a time-dependent random effects PRF model. The corresponding sample configuration formulas of the proposed theoretical model are applied to a set of 91 *Drosophila* genes in two species of African populations, *melanogaster* and *simulans* [33]. The main inferences are that i) the majority of newly-arisen nonsynonymous mutations that have been observed as polymorphism or divergence within *Drosophila* species are mildly deleterious with a mean selection coefficient of -2.81 times the reciprocal of the haploid effective population size, ii) almost 86% of the fixed differences between species are driven by positive selection, and iii) the estimated species divergence time between *D. melanogaster* and *D. simulans* is 1.72 million years ago. Two sets of simulated polymorphism and divergence data with 30 genes each were applied to the proposed model to check the validity of the MCMC simulation algorithm developed for the model.

## Materials and methods

### A Time-dependent random effects model

At any one locus, consider a sample of size *m* of aligned coding sequences from one species and another sample of size *n* of the orthologous sequences from a closely related species. We assume that the two species are so close that multiple mutations at the same site are negligible. The nucleotide sites that are polymorphic across the two samples can be classified into one of the following four categories: silent fixed differences (synonymous sites that are monomorphic within the two samples but different between them), silent polymorphisms (synonymous sites that are polymorphic in one or both samples), replacement fixed differences (nonsynonymous sites that are monomorphic within both samples but different between the samples), or replacement polymorphisms (nonsynonymous sites that are polymorphic in one or both samples). The McDonald and Kreitman (MK) 2 × 2 contingency table is composed of the above four types of counts. In the original time-independent PRF model of Sawyer and Hartl, the four counts of the MK table were described as four independent Poisson random variables whose expected values are calculated from the fixation flux and limiting distribution of polymorphic nucleotide substitutions [14]. For the time-dependent case, the MK table was generalized to a 2 × 3 contingency table by reclassifying the polymorphic sites into new polymorphic sites (sites that are polymorphic in only one sample) or legacy polymorphic sites (sites that are polymorphic in both samples) [29].

We assume that the two species have an equal and constant haploid effective population size *N*_*e*_ as their common ancestor and they have diverged *t*_div_*N*_*e*_ generations ago. At each locus, let *θ*_*s*_ and *θ*_*r*_ represent the rates of mutations to synonymous and nonsynonymous nucleotides that are likely to becoming polymorphic or fixed and *γ* the selection coefficient of a nonsynonymous mutation. These parameters are scaled in terms of the haploid effective population size so that *γ* = *N*_*e*_*s* and *θ* = *N*_*e*_*μ*, with *s* and *μ* being the conventional selection coefficient and mutation rate. Assuming that all synonymous mutations are selectively neutral (that is *γ* = 0), our goal is to estimate the distribution of the selection coefficient *γ* and the species divergence time *t*_div_. Using diffusion approximation to discrete time discrete state Markov chain, the distribution of site polymorphisms in a limiting infinitely large random mating population can be modeled as Poisson random fields [29]. Moreover, the theoretical results at population level were used to derive the distributions of the six counts in the generalized 2 × 3 contingency table. Under the assumption that nucleotide sites evolve independently, the six counts are independent Poisson random variables with expected values depending on the scaled population parameters *γ*, *θ*_*s*_, *θ*_*r*_, and *t*_div_. Mathematical derivation of the expected values are given in [29]. Now, suppose that values of the selection coefficient *γ*, at the *i*^*th*^ locus, is normally distributed with mean *γ*_*i*_ and variance $\sigma_{w}^{2}$ and values of the *γ*_*i*_ across all loci is normally distributed with mean *μ*_*γ*_ and variance $\sigma_{b}^{2}$.

In an aligned DNA sequences of one genetic locus, say locus *i*, from two closely related species, the expected values of the replacement (or nonsynonymous) fixed differences *K*_*ri*_, the replacement new polymorphisms *O*_*ri*_, and the replacement legacy polymorphisms *H*_*ri*_ are given by

$$\begin{array}{c} E\left(K_{ri}\right)=\frac{\theta_{r}}{s(1)}\int_{-\infty }^{\infty }N\left(\gamma |\gamma_{i},\sigma_{w}\right)\Lambda_{1}\left(\gamma ,t_{\text{div}},m,n\right)d\gamma \end{array}$$ (1)

$$\begin{array}{c} E\left(O_{ri}\right)=\frac{\theta_{r}}{s(1)}\int_{-\infty }^{\infty }N\left(\gamma |\gamma_{i},\sigma_{w}\right)\Lambda_{2}\left(\gamma ,t_{\text{div}},m,n\right)d\gamma \end{array}$$ (2)

$$\begin{array}{c} E\left(H_{ri}\right)=\frac{\theta_{r}}{s(1)}\int_{-\infty }^{\infty }N\left(\gamma |\gamma_{i},\sigma_{w}\right)\Lambda_{3}\left(\gamma ,t_{\text{div}},m,n\right)d\gamma , \end{array}$$ (3)

where *N*(*γ*|*γ*_*i*_, *σ*_*w*_) represents the probability density function of a normal random variable with mean *γ*_*i*_ and variance $\sigma_{w}^{2}$ and

$$\begin{array}{ccc} \Lambda_{1}\left(\gamma ,t_{\text{div}},m,n\right) & = & \int_{0}^{1}(I(x,m)K(x,n)+I(x,n)K(x,m))(s(1)\;-\;s(x))\;m\left(dx\right)\\ & + & 2(t_{\text{div}}\;-\;\int_{0}^{t_{\text{div}}}\int_{0}^{1}(\lim_{x\to 0}\frac{p(u,x,y)}{s(x)})s\left(y\right)m\left(dy\right)du)+L\left(m\right)+L\left(n\right) \end{array}$$ (4)

$$\begin{array}{ccc} \Lambda_{2}\left(\gamma ,t_{\text{div}},m,n\right) & = & \int_{0}^{1}(2\;-\;x^{m}\;-\;{\left(1-x\right)}^{m}\;-\;x^{n}\;-\;{\left(1-x\right)}^{n}\\ & & -2J\left(x,m\right)J\left(x,n\right))(s(1)\;-\;s(x))\;m\left(dx\right) \end{array}$$ (5)

$$\begin{array}{ccc} \Lambda_{3}\left(\gamma ,t_{\text{div}},m,n\right) & = & \int_{0}^{1}J\left(x,m\right)J\left(x,n\right)(s(1)-s(x))\;m\left(dx\right). \end{array}$$ (6)

In the above expressions *I*(*x*, *m*), *J*(*x*, *m*), and *K*(*x*, *m*) denote respectively the probability that a nucleotide site is monomorphic in the sample at the wild-type (non-mutant), the probability that the site is polymorphic in the sample, and the probability that the site is monomorphic at the mutant nucleotide. Their specific expressions are given by

$$\begin{array}{ccc} & I(x,m)= & \frac{s(1)-s(x)}{s(1)}-\int_{0}^{1}p\left(t_{\text{div}},x,y\right)(1-{\left(1-y\right)}^{m}-\frac{s(y)}{s(1)})\;m\left(dy\right)\\ & J(x,m)= & \int_{0}^{1}p\left(t_{\text{div}},x,y\right)(1-y^{m}-{\left(1-y\right)}^{m})\;m\left(dy\right)\\ & K(x,m)= & \frac{s(x)}{s(1)}+\int_{0}^{1}p\left(t_{\text{div}},x,y\right)(y^{m}-\frac{s(y)}{s(1)})\;m\left(dy\right). \end{array}$$

Also

$$\begin{array}{c} L\left(m\right)=\int_{0}^{1}x^{m}(s(1)-s(x))\;m\left(dx\right)-\int_{0}^{1}\int_{0}^{1}p\left(t_{\text{div}},x,y\right)y^{m}(s(1)-s(x))\;m\left(dy\right)m\left(dx\right) \end{array}$$

The functions *s*(*x*) and *m*(*dx*) appeared in Eqs (1)–(6) are called the scale function and speed measure of the limiting diffusion process and defined by *s*(*x*) = (1 − *e*^−*γx*^)/*γ* and *m*(*dx*) = *e*^*γ*^*dx*/(*x*(1 − *x*)) for replacement sites and *s*(*x*) = *x* and *m*(*dx*) = *dx*/(*x*(1 − *x*)) for silent sites (i.e. *γ* = 0). The transition probability density *p*(*t*, *x*, *y*) satisfies that for any continuous function *f*(*x*) on [0, 1], the integral $u\left(t,x\right)=\int_{0}^{1}p\left(t,x,y\right)f\left(y\right)m\left(dy\right)$ is the solution of the diffusion equation

$$\begin{array}{c} \frac{\partial u(t,x)}{\partial t}=x\left(1-x\right)\frac{\partial^{2}u\left(t,x\right)}{\partial x^{2}}+\gamma x\left(1-x\right)\frac{\partial u(t,x)}{\partial x} \end{array}$$ (7)

for *t* > 0 and 0 < *x* < 1, with

$$\begin{array}{c} u(t,0)=u(t,1)=0\;u(0,x)=f(x) \end{array}$$ (8)

Similarly, the expected values of the silent (or synonymous) fixed differences *E*(*K*_*si*_), silent new polymorphisms *E*(*O*_*si*_), and silent legacy polymorphisms *E*(*H*_*si*_) are given by Eqs (1)–(3) with *γ* = 0.

### Adaptive directional adaptive Metropolis MCMC sampling algorithm

For a set of *L* loci, the model contains three types of within-locus parameters *θ*_*ri*_, *θ*_*si*_, and *γ*_*i*_, *i* = 1, 2, …, *L* as well as four across-loci parameters *t*_div_, *μ*_*γ*_, *σ*_*b*_, and *σ*_*w*_. These parameters can be estimated by Markov chain Monte Carlo simulations under a hierarchical Bayesian framework. Specifically, we use gamma distributions with given parameters as prior distributions of the two types of mutation rates, *θ*_*si*_, *θ*_*ri*_, a normal-inverse-gamma distribution as a conjugate prior of the mean *μ*_*γ*_ and between-locus variance $\sigma_{b}^{2}$, and uniform distributions for the divergence time *t*_div_ and within-locus standard deviation *σ*_*w*_. That is

$$\begin{array}{ccc} \theta_{s,i} & ∼ & \Gamma (\alpha_{s},\beta_{s})\\ \theta_{r,i} & ∼ & \Gamma (\alpha_{r},\beta_{r})\\ \left(\mu_{\gamma },\sigma_{b}\right) & ∼ & NIG(\alpha_{0},\beta_{0},\mu_{0},n_{0})\\ t_{\text{div}} & ∼ & U(0,t_{\text{max}})\\ \sigma_{w} & ∼ & U(0,\sigma_{\text{max}}) \end{array}$$ (9)

All hyperparameters *α*_0_, *β*_0_, *α*_*s*_, *β*_*s*_, *α*_*r*_, *β*_*r*_, *μ*_0_, and *n*_0_ are chosen to be small (∼ 0.001) so as to be “uninformative” and *t*_max_ and *σ*_max_ are large fixed values. Based on the sampling formulas given by Eqs (1)–(3) and the prior distributions given by Eq (9), a joint posterior distribution of the model parameters can be written as

$$\begin{array}{ccc} & & L(\theta_{si},\theta_{ri},\mu_{\gamma },\sigma_{b},\gamma_{i},\sigma_{w},t_{\text{div}},K_{si},O_{si},H_{si},K_{ri},O_{ri},H_{ri})\\ & & =\prod_{i=1}^{L}\{N\left(\gamma_{i}|\mu_{\gamma },\sigma_{b}\right)\Gamma \left(\theta_{si}|\alpha_{s},\beta_{s}\right)\Gamma \left(\theta_{ri}|\alpha_{r},\beta_{r}\right)\\ & & \times {\text{Poi}}_{1}\left(\theta_{si},0,0,t_{\text{div}},K_{si},m_{i},n_{i}\right){\text{Poi}}_{2}\left(\theta_{si},0,0,t_{\text{div}},O_{si},m_{i},n_{i}\right)\\ & & \times {\text{Poi}}_{3}\left(\theta_{si},0,0,t_{\text{div}},H_{si},m_{i},n_{i}\right){\text{Poi}}_{1}\left(\theta_{ri},\gamma_{i},\sigma_{w},t_{\text{div}},K_{ri},m_{i},n_{i}\right)\\ & & \times {\text{Poi}}_{2}\left(\theta_{ri},\gamma_{i},\sigma_{w},t_{\text{div}},O_{ri},m_{i},n_{i}\right){\text{Poi}}_{3}\left(\theta_{ri},\gamma_{i},\sigma_{w},t_{\text{div}},H_{ri},m_{i},n_{i}\right)\}\\ & & \times \;\Gamma \left(\frac{1}{\sigma_{b}^{2}}|\alpha_{0},\beta_{0}\right)N\left(\mu_{\gamma }|\mu_{0},\frac{\sigma_{b}}{\sqrt{n_{0}}}\right)u\left(t|0,t_{\text{max}}\right)u\left(\sigma_{w}|0,\sigma_{\text{max}}\right) \end{array}$$ (10)

where *L* is the total number of loci, *N*(*y*|*μ*, *σ*), Γ(*y*|*α*, *β*) and *u*(*y*|0, *Y*) are respectively normal, gamma and uniform probability densities, and

$$\begin{array}{c} {\text{Poi}}_{j}\left(\theta ,\gamma ,\sigma_{w},t_{\text{div}},c_{j},m,n\right)=\frac{e^{-\lambda_{j}}{\left(\lambda_{j}\right)}^{c_{j}}}{c_{j}!}\;j=1,2,3; \end{array}$$

where

$$\begin{array}{c} \left\{ \begin{array}{c} c_{1}=K_{s},\lambda_{1}=E\left(K_{s}\right)\;\text{or}\;c_{1}=K_{r},\lambda_{1}=E\left(K_{r}\right)\\ c_{2}=O_{s},\lambda_{2}=E\left(O_{s}\right)\;\text{or}\;c_{2}=O_{r},\lambda_{2}=E\left(O_{r}\right)\\ c_{3}=H_{s},\lambda_{3}=E\left(H_{s}\right)\;\text{or}\;c_{3}=H_{r},\lambda_{3}=E\left(H_{r}\right) \end{array}\right. \end{array}$$

In general, at each step of the Monte Carlo simulations, the two types of the mutation rates *θ*_*ri*_ and *θ*_*si*_ are updated by Gibbs-samplers based on gamma distributions and the selection coefficient *γ*_*i*_ is updated by Metropolis random-walk algorithm. Upon finish of the above process for all of the *L* loci, two global parameters *μ*_*γ*_ and *σ*_*b*_ are updated from a normal-inverse-gamma distribution according to a Gibbs-sampler and the other two global parameters *t*_div_ and *σ*_*w*_ are updated individually using two Metropolis random-walks.

However, the practice of the above described sampling method was unsuccessful in the sense that the underlying Markov chains did not converge or converged extremely slow to their target distributions. The reason for the slow convergence is that each of the three parameters (*μ*_*γ*_, *σ*_*b*_, *σ*_*w*_) has a high autocorrelation which makes proposal values rely heavily on previous values and hence the chain moves slowly through entire parameter space. Although, in theory, the chain will eventually converge to its stationary distribution in a long iteration, a more approachable solution is to improve the proposal distribution of the Metropolis algorithm. Haario et al. proposed an adaptive Metropolis (AM) algorithm to adjust both the step size and spatial orientation of an assumed Gaussian proposal distribution [34]. Application of the AM algorithm did reduce the autocorrelation but the sampling efficiency is still low due to the existence of high correlation among (*μ*_*γ*_, *σ*_*b*_, *σ*_*w*_).

It is quite common that MCMC simulations in high dimension, like the current situation, introduce significant amount of correlation among parameters and hence the searching paths are sometimes dominated by some of the parameters. As the result, the sampling trajectories will be trapped at a rather restricted area of the whole parameter space. Bai Proposed an adaptive directional Metropolis-within-Gibbs (ADMG) algorithm to adjust both sampling direction and scale componentwisely with a Metropolis-within-Gibbs sampler [35]. Here we adopted both AM and ADMG algorithms to propose an adaptive directional adaptive Metropolis (ADAM) algorithm to update the three parameters (*μ*_*γ*_, *σ*_*b*_, *σ*_*w*_) jointly. Based on the algorithm, a singular value decomposition (SVD) is performed on the empirical covariance matrix and orthonormal vectors from the SVD are used as sampling directions. Specifically, in a total of 2,000,000 iterations, we first run the above described process for 50,000 iterations to obtain an empirical variance covariance matrix of (*μ*_*γ*_, *σ*_*b*_, *σ*_*w*_), say *C*_0_. The three parameters were then jointly updated for another 100,000 iterations using a multivariate normal distribution with the fixed covariance matrix *C*_0_. At each step of the final 1,850,000 iterations, we (1) performed a singular value decomposition on the empirical covariance matrix *C*_*t*_, *t* > 150,000 such that *C*_*t*_ = *D*Σ_*t*_*D*^*T*^, (2) set $Y_{t}=D^{T}X_{t}^{T}$, (3) updated *Y*_*t*_ based on a three-dimensional normal distribution with mean *Y*_*t*_ and variance-covariance matrix *δ*Σ_*t*_, (4) transformed *Y*_*t*_ back to the original set of parameters by (*D*^*T*^)^−1^*Y*_*t*_, (5) and recalculated the empirical covariance matrix recursively. Here *δ* = exp(2*d*(*δ*^(*k*)^ − 0.3)) is a jumping scale, *d* = 3 is the dimension of the vector of parameters and *δ*^(*k*)^ is an average acceptance rate for every *k* iterations with *k* = 100 in our implementation. The resulting chain from above updating process is no longer Markovian due to the fact that calculation of the empirical covariance matrix uses cumulative information from all previous states. However, it can be shown that the chain with adapted direction satisfies both the diminishing adaptive condition and the bounded convergence condition and hence it would converge to the target distribution [36]. In the calculation of the three Poisson means, given by Eqs (1)–(3), Crank-Nicholson method was used to integrals involving the transition density *p*(*t*, *x*, *y*), Gauss-Legendre quadrature was used to numerically solve integrals from 0 to 1, and Gauss-Hermit quadrature was used for integrations over (−∞, +∞) [37]. The whole updating procedure was implemented using a parallel computing technique, Message Passing Interface (MPI) [38]. It is noticed that leg-5 autocorrelations for *μ*_*γ*_, *σ*_*b*_ and *σ*_*w*_ range from −0.01 to 0.34 across the two simulated data sets as well as the set of 91 *Drosophila* genes showing that the proposed sampling algorithm could be useful in MCMC simulations where model parameters are highly auto-correlated.

## Results

### Simulation study

**Fig 1:**
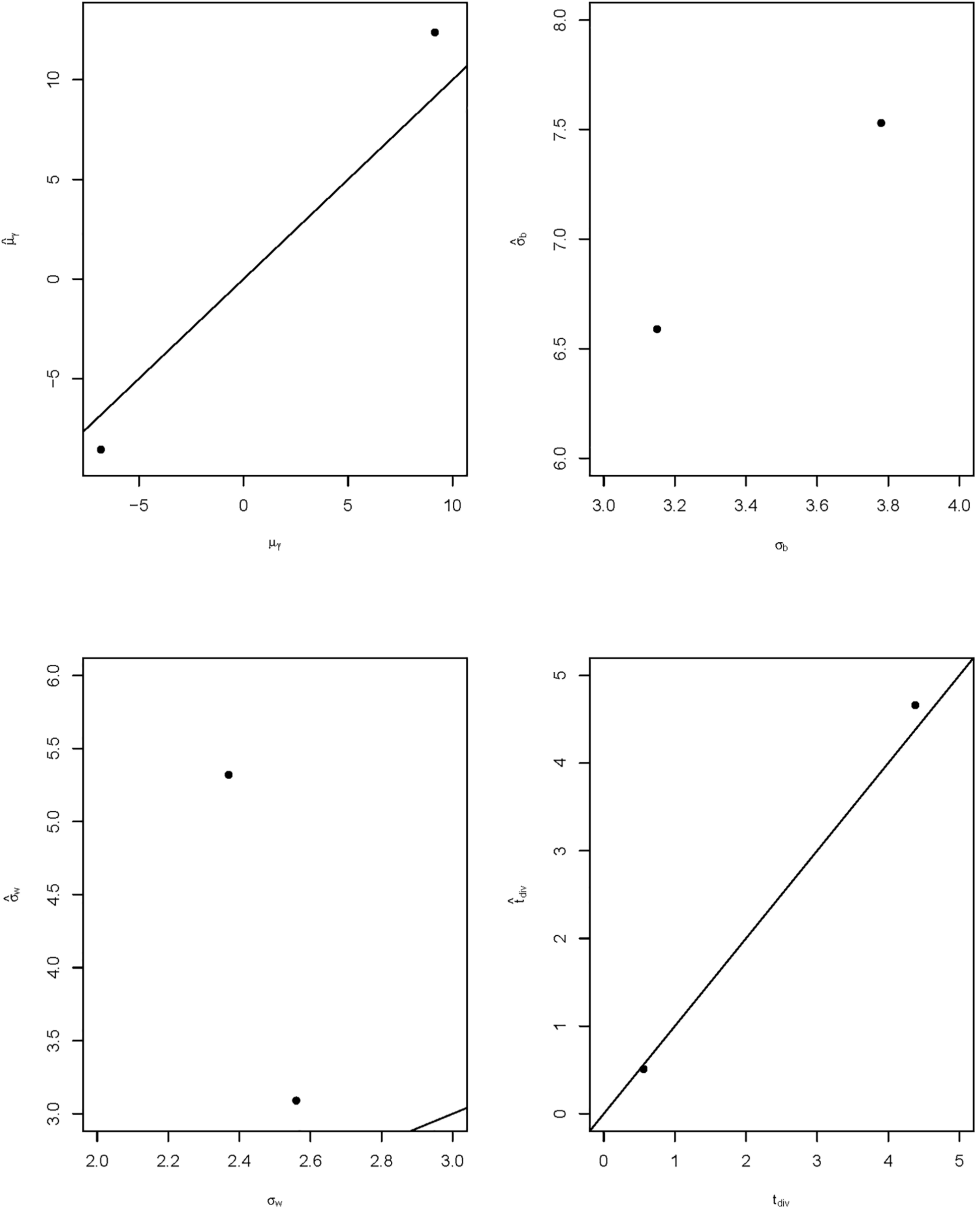
True vs. estimated values of the four model parameters. The true values (x-axes) of the four parameters (*μ*_*γ*_, *σ*_*b*_, *σ*_*w*_, *t*_div_) and their corresponding model estimates (y-axes) (${\hat{\mu }}_{\gamma },{\hat{\sigma }}_{b},{\hat{\sigma }}_{w},{\hat{t}}_{\text{div}}$) for the two simulated data sets. Straight lines represent *y* = *x*.

Two data sets each containing 30 loci were generated according to the following three steps. First, the four global parameters *μ*_*γ*_, *σ*_*b*_, *σ*_*w*_, and *t*_div_ were set to be fixed. Second, at each locus, the silent and replacement mutation rates *θ*_*s*_ and *θ*_*r*_ were generated from two continuous uniform distributions with given ranges, the numbers of alignment sequences *m* and *n* were drawn from two discrete uniform distributions with certain ranges, and the selection coefficient *γ* was sampled from a normal distribution with mean *γ*_*m*_ and variance $\sigma_{w}^{2}$, where *γ*_*m*_ was a random draw from a normal distribution with mean *μ*_*γ*_ and variance $\sigma_{b}^{2}$. Third, the six counts of a locus specific 2 × 3 contingency table were obtained from Poisson distributions where the expected values are given by Eqs (1)–(3) for replacement sites and Eqs (1)–(3) with *γ* = 0 for silent sites. Specifically, the given values of the parameters (*μ*_*γ*_, *σ*_*b*_, *σ*_*w*_, *t*_div_) for the two simulated data sets are (−6.82, 3.78, 2.56, 4.38) and (9.15, 3.15, 2.37, 0.56) respectively. After disregarding the first 250,000 iterations as a burn-in period, 5,000 samples were taken every 400 steps to form ten consecutive subchains. Convergence of the chain is confirmed by trace plots and Gelman-Rubin (GR) diagnostic being less than 1.1 [39]. The median estimates of the above parameters from last subchains are (−8.56, 7.53, 3.09, 4.66) for the first data set and (12.38, 6.59, 5.32, 0.51) for the second set. The true values of the four parameters (*μ*_*γ*_, *σ*_*b*_, *σ*_*w*_, *t*_div_) and those estimated from the proposed model for the two simulated data sets are plotted in Fig 1. For both data sets, the divergence time *t*_div_ converged quickly to their true values with slight variation but most of the simulation results tend to overestimate the selection parameters *μ*_*γ*_, *σ*_*b*_ and *σ*_*w*_. The magnitude of the estimated mean selection coefficient ${\hat{\mu }}_{\gamma }$ in both data sets was approximately 1.3 times larger than their corresponding true values but the sign of the parameter stayed the same as the given values. Estimates of the between-locus variance ${\hat{\sigma }}_{b}$ for the two simulated data sets were roughly twice as large as the given values and hence the scatter plot of *σ*_*b*_ versus ${\hat{\sigma }}_{b}$ in Fig 1 could not show the diagonal line of *y* = *x*. Similarly, the estimates of the within species variance ${\hat{\sigma }}_{w}$ for the two simulated data sets were 1.2 and 2.2 times larger than their given values. The 95% credible intervals of the four parameters all covered the given values. One possible reason for such behavior is that *σ*_*b*_ and *σ*_*w*_ are two artificial parameters implanted into the model to be biologically realistic but they are lack of data support. It may require a much longer MCMC simulation runs to capture the true values of *σ*_*b*_ and *σ*_*w*_ or adding more loci into the data may supply more information about the between and within loci variations.

### Results on polymorphism and divergence data from Drosophila

**Fig 2:**
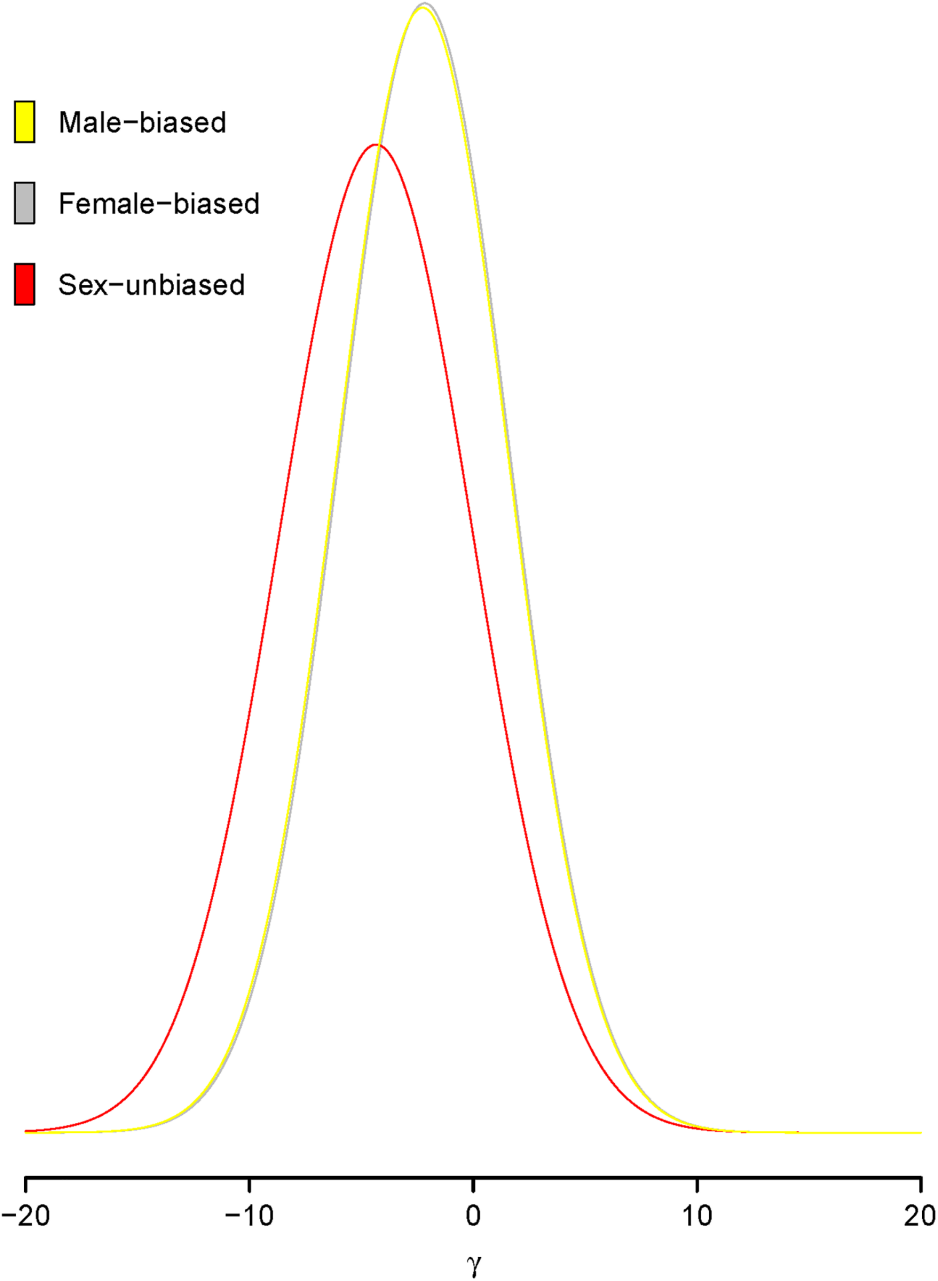
Distribution of selective effects. Estimated distribution of scaled selection coefficients *γ* of newly arisen nonsynonymous mutations that have been observed as polymorphism or divergence within *Drosophila* species. The distributions infer only for those mutations whose selective effects are not so severe such that there is a reasonable chance for these mutations to accumulate high frequencies in a population and hence to be included in a relatively small sample. Three distributions are based on the estimates of the 33 male-biased genes (yellow), 28 female-biased genes (gray), and 30 sex-unbiased genes (red).

**Fig 3:**
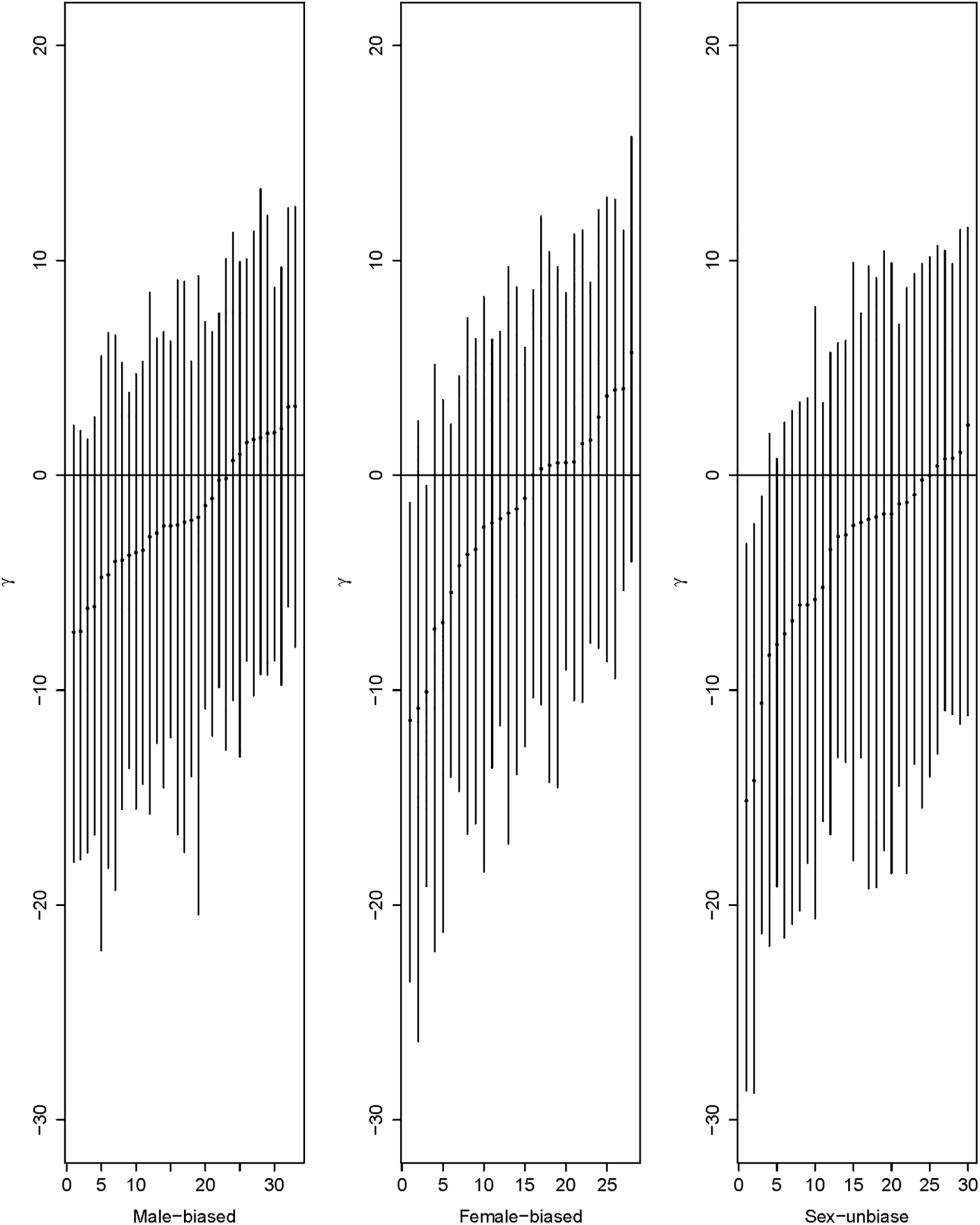
Estimated selection coefficients for the three gene classes. Median estimates of the scaled selection coefficient *γ* for the male-biased, female-biased, and sex-unbiased genes with the loci sorted by the values of the estimates. Error bars represent 95% credible intervals.

The time-dependent random effects PRF model was applied to the data of [33]. The data contains the coding sequences of 91 genes in samples of *Drosophila melanogaster* collected from Lake Kariba, Zimbabwe [40]. The number of alignments of the DNA sequences ranges from seven to twelve. As a comparison of the intraspecific polymorphism with interspecific divergence, a single highly inbred line of *Drosophila simulans* was sampled from Chapel Hill, North Carolina [41]. These 91 genes were classified as male-biased (33 out of the 91), female-biased (28 out of the 91) and sex-unbiased (30 out of the 91) genes based on the difference of gene expression level between testes and ovaries. After 150,000 burn-in iterations, ten subchains were formed by taking samples every 400 steps to reduce autocorrelation. Each subchain contains 500 samples and model parameters were estimated using median values and their 95% credible intervals (CIs) from last subchain. In diffusion time scale, for all 91 genes together, the mean selection coefficient *μ*_*γ*_ = −2.81 with a 95% CI of (−9.71, 2.68), the between-loci standard deviation *σ*_*b*_ = 6.00 with (3.27, 9.09), the within-locus standard deviation *σ*_*w*_ = 6.16 with (0.39, 9.76), and the species divergence time *t* = 2.67 with a 95% CI of (2.48, 2.89). This estimated negative mean selection coefficient supports the viewpoint that most newly arisen nonsynonymous mutations are deleterious [22–24, 42, 43]. The same data was applied to a mutation-selection-drift equilibrium random effects PRF model and estimated a mean selection coefficient of −5.7 based on 21,000,000 MCMC iterations [24], while application of the same data to a time-dependent fixed effects model gave an estimate of 1.98 for *μ*_*γ*_ [30]. Although it is biologically more realistic to model selective effects within a gene as a random variable, as in [24], assuming mutation-selection-drift equilibrium may bias estimates of the selective effects. On the other hand, building the species divergence time explicitly into a model, as in [30], is less artificial but the assumption of constant selection within a gene may fail to capture negative selective effects. When we apply the proposed random effects model individually to the three expression classes of genes, the estimated mean selection coefficients and their 95% credible intervals for the 33 male-biased genes, 28 female-biased genes and the 30 sex-unbiased genes are respectively −2.27 with (−9.10, 3.18), −2.17 with (−8.54, 3.61) and −4.34 with (−11.95, 1.95). The distributions of the scaled selection coefficients for the three groups of genes are presented in Fig 2, expressed in terms of normal density curves. The three density curves in Fig 2 are quite similar to those in Sawyer et al. [24] except that the magnitude of the mean values based on our proposed time-dependent random effects model is smaller than the estimated mean values using time-independent random effects model given in [24]. It is likely that the artificial assumption of mutation-selection-drift equilibrium in [24] biased the estimates of the selection coefficients. Using median estimates and their corresponding 95% credible intervals, Fig 3 shows the selection coefficients of individual genes for the three expression classes with the loci sorted by the values of the estimates. Based on the estimates, 30% of male-biased and 46% of female-biased genes are under positive selection while only 16.6% of the nonsynonymous mutations observed in sex-unbiased genes are beneficial. Our finding suggests that newly arisen replacement mutations in sex-biased genes are more likely to be beneficial. However, the nonsynonymous mutations in Figs 3 and 2 include only those mutations whose deleterious effects are not very severe so that there is a reasonable chance for these mutations to accumulate high frequencies in a population and hence to be included in a relatively small sample.

If we assume that *N*_*e*_ generations is 0.645 million years for *Drosophila* [14], the estimated *t* = 2.67 implies a species divergence time of 1.72 million years between *D. melanogaster* and *D. simulans*. This value falls almost in the middle of the range 0.8–3 million years, which has been used as a standard of comparison [44, 45]. When the time-dependent random effects model was individually applied to the 33 male-biased genes, 28 female biased genes and the 30 sex-unbiased genes to estimate selection parameters, the model also generated estimates for the divergence time parameter *t*_div_. It turns out that the three estimates from the three expression classes are identical to the estimated *t*_div_ using the 91 genes together, which shows that the proposed time-inhomogeneous random effects model is biologically realistic.

**Fig 4:**
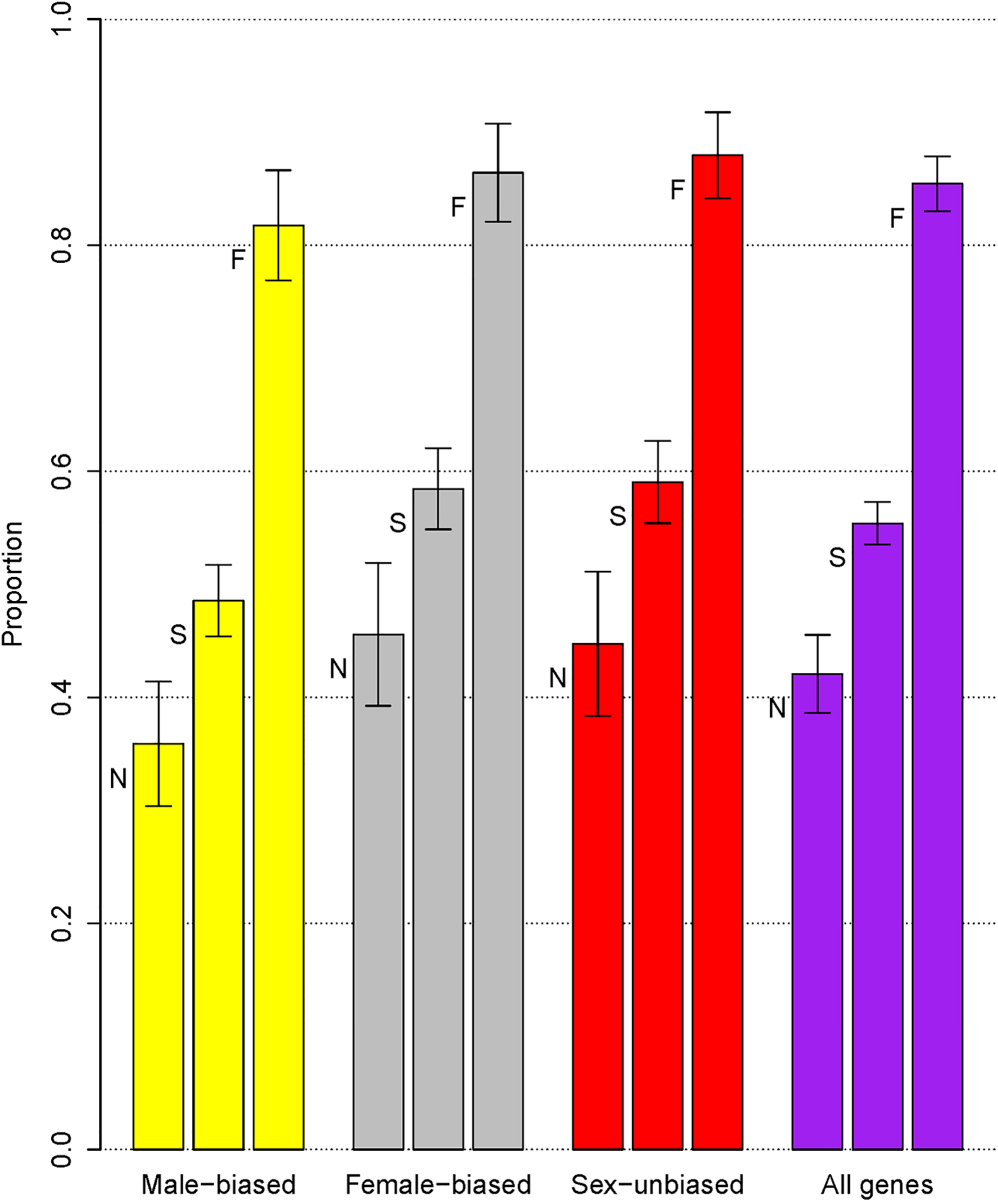
Estimates of the expected population proportions. Median estimates of the expected population proportions of positively selected nonsynonymous mutations among newly arisen new mutations (N), sample polymorphisms (S), and sample fixed differences (F) with error bars representing 95% credible intervals. Proportions are calculated based on the 33 male-biased genes (yellow), 28 female-biased genes (gray), 30 sex-unbiased genes (red), and the 91 genes together (purple).

One distinguishing feature of the random effects model is its ability to estimate important quantities in the area of population genetics such as the expected population proportion of nonsynonymous substitutions that are positively selected among new mutations, the expected population proportion of nonsynonymous substitutions that are positively selected among polymorphisms present in the sample, and the positively selected population proportion among fixed differences between the species. The expected population proportions of the beneficial new mutations at each locus is given by the following integral

$$\begin{array}{c} \int_{0}^{+\infty }N\left(\gamma |\gamma_{i},\sigma_{w}\right)\;d\gamma \end{array}$$

and the estimates of the quantity across the 91 genes are low, with a median value of 0.421. The expected population proportions of sample polymorphisms due to positive selection at each locus, estimated as

$$\begin{array}{c} \frac{\int_{0}^{+\infty }(\Lambda_{2}\left(\gamma ,t,m,n\right)+\Lambda_{3}\left(\gamma ,t,m,n\right))N\left(\gamma |\gamma_{i},\sigma_{w}\right)\;d\gamma }{\int_{-\infty }^{+\infty }(\Lambda_{2}\left(\gamma ,t,m,n\right)+\Lambda_{3}\left(\gamma ,t,m,n\right))N\left(\gamma |\gamma_{i},\sigma_{w}\right)\;d\gamma } \end{array}$$

are higher for the 91 genes, with a median value of 0.554. The expected population proportions of fixed differences due to positive selection at each locus, calculated by

$$\begin{array}{c} \frac{\int_{0}^{+\infty }\Lambda_{1}\left(\gamma ,t,m,n\right)N\left(\gamma |\gamma_{i},\sigma_{w}\right)\;d\gamma }{\int_{-\infty }^{+\infty }\Lambda_{1}\left(\gamma ,t,m,n\right)N\left(\gamma |\gamma_{i},\sigma_{w}\right)\;d\gamma } \end{array}$$

are significantly higher, with a median value of 0.854. The functions Λ_1_, Λ_2_ and Λ_3_ are defined in Eqs (4)–(6). The three types of population proportions for all 91 genes together as well as individually for the male-biased, female-biased and sex-unbiased genes are displayed in Fig 4 and the results are quite consistent with those obtained from a time-homogeneous random effects model [24].

## Discussion

We have developed a Poisson random field model for estimating the distribution of selective effects of newly arisen mutations that could be observed as polymorphism or divergence in samples of two related species and species divergence time under the assumption that the two species populations are not at mutation-selection-drift equilibrium. One limitation of our Bayesian random effects model is the assumption of constant and equal population sizes of the two daughter species and their common ancestor. Certain types of demographic changes may influence the fate of mutant alleles and ignorance of such changes can confound the interpretation of polymorphism and divergence and hence results in biased estimates of the selective effects [4, 12, 21, 43, 46, 47]. For instance, some classes of deleterious nonsynonymous polymorphisms which might previously have remained polymorphic could be eliminated due to a sudden increase in the effective population size and thereby causing a decrease of the nonsynonymous polymorphisms without affecting nonsynonymous divergence. Although the *Drosophila melanogaster* data applied in our study was derived from African populations that have relatively less demographic complexity [40, 48], a more sophisticated model that takes into account various demographic changes while inferring natural selection is need to be developed. Williamson et al. proposed a time-inhomogeneous PRF model to make inference about constant selection and population growth simultaneously based on Single Nucleotide Polymorphism (SNP) data from one species [25]. Boyko et al. extended the site frequency spectrum based PRF approach to allow for simultaneous inference of demography and the distribution of fitness effects among newly arisen mutations [21]. The differences between our approach and theirs are that their studies are based on maximum likelihood methods and applied to site frequency spectrum data from single population. Simulation results have shown that PRF models with genic selection can strongly bias the estimates of selection parameters when the underlying data is a frequency spectrum of polymorphisms from one population but the estimates are nearly unbiased for the polymorphism and divergence data from two related species [27].

Our model also assumes that nucleotide sites at each genetic locus evolve independently while the various local rate of recombination tells us that the nucleotides within a gene are more or less linked. As for estimating the mean selection coefficient, simulation results have shown that PRF approaches are relatively robust to violation of independent site assumption [16, 21, 28]. Nevertheless, inferences about the distributions of the selective effects for tightly linked genes based on PRF models should still be interpreted cautiously. At a particular locus, the distribution of selective effects of nonsynonymous mutations that have become polymorphic or fixed in a sample is assumed to be Gaussian which has fixed variance across loci. The normal assumption in a continuous time model of selection is natural based on the Central Limit Theorem [10]. Other alternatives that have been considered include some heavy-tailed distributions such as Laplace and Chi-square [49], nearly exponentially distribution [50] or gamma distribution with a shape parameter between 0.1 and 1 [51].

To what degree the genetic variation observed in a polymorphism and divergence data links to phenotypic variation, especially to those medically interesting phenotypes are unclear [52–55]. It seems plausible that some rare and negatively selected nonsynonymous mutations are related to certain human genetic diseases and hence our estimates of the distribution of the selective effects may help identifying genes that might have related to underlying diseases. In fact, we have applied the time-dependent random effects PRF model to a data containing coding sequences of whole genome of two patients with cytogenetically normal myelodysplatic syndrome (CN-MDS). Based on our preliminary estimates from chromosome one, there are about 33 genes whose scaled selection coefficients are smaller than -20, about 230 genes with −20 < *γ* < −10, and 160 genes whose *γ* values are bigger than -10 but smaller than zero. Of course, these results based on our current model are very rough references for disease gene identification and a model which will be more suitable for the application of polymorphism and divergence data from cancer patients and healthy population is under development.

## Supporting information

S1 File Data of the 91 Drosophila genes. The data contains the coding sequences of 91 genes in samples of *Drosophila melanogaster* collected from Lake Kariba, Zimbabwe [40] and a single highly inbred line of *Drosophila simulans* from Chapel Hill, North Carolina [41]. In the file, Column 1 and 2 list the numbers of alignments of the DNA sequences from the two species (M and N). Column 3-8 are the numbers of silent fixed difference (Sf), silent new polymorphism (Snp), silent legacy polymorphism (Slp), replacement fixed difference (Rf), replacement new polymorphism (Rnp) and replacement legacy polymorphism (Rlp). Column 9 (Locus) lists the names of the genes and the last column (Class) classifies these 91 genes as male-biased (M), female-biased (F) and sex-unbiased (U) genes based on the difference of gene expression level between testes and ovaries.
(TXT)
